# Composition of the murine gut microbiome impacts humoral immunity induced by rabies vaccines

**DOI:** 10.1002/ctm2.161

**Published:** 2020-08-22

**Authors:** Yachun Zhang, Qiong Wu, Ming Zhou, Zhaochen Luo, Lei Lv, Jie Pei, Caiqian Wang, Benjie Chai, Baokun Sui, Fei Huang, Zhen F. Fu, Ling Zhao

**Affiliations:** ^1^ State Key Laboratory of Agricultural Microbiology Huazhong Agricultural University Wuhan China; ^2^ Key Laboratory of Preventive Veterinary Medicine of Hubei Province College of Veterinary Medicine Huazhong Agricultural University Wuhan China

**Keywords:** Clostridia, gut microbiome, humoral immunity, Lachnospiraceae, rabies virus, virus‐neutralizing antibody

## Abstract

**Background:**

Gut microbiome plays a crucial role in modulating human and animal immune responses. Rabies is a fatal zoonosis causing encephalitis in mammals and vaccination is the most effective method to control and eliminate rabies. The relationship between the gut microbiome and humoral immunity post rabies vaccination has not been investigated yet.

**Methods:**

Mice orally administrated with a cocktail of broad‐spectrum antibiotics were inoculated with rabies vaccines, and humoral immune response was analyzed at indicated time points. The 16S ribosomal RNA (16S rRNA) gene sequencing was performed on fecal samples from groups in vancomycin‐treated and untreated mice. Mice were immunized with rabies vaccines and virus‐neutralizing antibody (VNA) levels were measured, resulting in VNA high (H) and low (L) groups. Then 16S rRNA gene sequencing was performed on fecal samples from H and L group mice.

**Results:**

After antibiotic (Abx) treatment, mice had decreased levels of rabies virus (RABV)‐specific IgM, IgG, and virus‐neutralizing antibody compared with untreated mice. Abx‐treated mice had fewer T follicular helper cells, germinal center B cells, and antibody secreting cells (ASCs) in lymph nodes than did untreated mice. Gut microbiome facilitated secondary immune responses by increasing the generation of ASCs. Treatment with vancomycin alone had a similarly impaired effect on the humoral immune responses compared with Abx‐treated mice. From the natural population group of mice received rabies vaccines, VNA titers vary significantly and the abundance of *Clostridiales* and *Lachnospiraceae* was positively associated with the antibody titers in mice.

**Conclusions:**

Our results provide the evidence that the gut microbiome impacts humoral immunity post rabies vaccination, and further investigation of the mechanism will help the development of novel adjuvants and vaccines.

## Graphical abstract

• Antibiotics treatment reduced the production of virus‐neutralizing antibody post rabies vaccination.

• Antibiotics treatment impaired the recruitment of T follicular helper cells, germinal center B cells, and antibody secreting cells post rabies vaccination.

• Vancomycin‐treatment impaired the rabies vaccine‐induced humoral immunity by decreasing several types of bacteria, including *Bacteroidia, Clostridia, Erysipelotrichia, Actinobacteria, Saccharimonadia*, and *Alphaproteobacteria*.

• The abundance of *Clostridiales* and *Lachnospiraceae* accounted for heterogenicity of humoral immunity in the natural population of mice immunized with rabies vaccines.

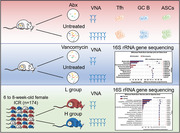

**Abbreviations**
ASCs: antibody secreting cells
BHK: baby hamster kidney
BSA: bovine serum albumin
CVS: challenge virus strain
DCs: dendritic cells
dLNs: draining lymph nodes
DMEM: Dulbecco's modified Eagle's medium
ELISA: enzyme‐linked immunosorbent assay
FAVN: fluorescent antibody virus neutralization
FFU: focus‐forming units
FITC: fluorescein isothiocyanate
GC: germinal center
GF: germ‐free
GVHD: graft‐versus‐host‐disease
LEfSe: LDA effect size
PBS: phosphate‐buffered saline
PCoA: principal coordinate analyses
qPCR: quantitative real‐time PCR
RABV: rabies virus
SAD: Street Alabama Dufferin
SCFAs: short‐chain fatty acids
Tfh: follicular helper T
TLR: Toll‐like receptor
TMB: tetra‐methyl‐benzidine
VNA: virus‐neutralizing antibody

## 1 BACKGROUND

The gut microbiome, the so‐called “second genome,” plays critical roles in regulating the host immunity.^1^, ^2^ Multiple studies have demonstrated the role of the gut microbiome in the regulation of human metabolism, obesity,^3^ tumorigenesis,^4^ enteric immunity,^5^ inflammatory bowel disease,^6^ allergic diseases,^7^ as well as its interplay with the gut‐brain axis,^8^ gut‐liver axis,^9^ and the gut‐lung axis.^10^ Interestingly, novel development coming with the emerging mechanism reveals the striking influence of gut microbiome on host's immune system.^1^

Rabies is a fatal zoonosis characterized by encephalitis and almost 100% mortality. Rabies causes more than 59 000 human deaths every year in worldwide, most of which occur in developing countries.^11^ Extensive efforts have been made to reduce these numbers, and massive vaccination of domestic animals is considered to be the most efficacious method to control and eliminate rabies.^12^ After a rabies vaccination, humoral immunity, especially the virus‐neutralizing antibodies, is the major immune effector against virulent RABV infection. Nevertheless, emerging evidences highlight the fact that the effect of a rabies vaccination varies among individuals, including humans.

Accumulating evidences indicate that the composition of the microbiome is of great importance in the regulation of immune responses.^13^ Immune responses to vaccines are likely to be mediated by various microbiome since the gut microbiome is closely related to the exploitation and maturity of the immune system.^14^ Previous research has demonstrated that the gut microbiome inhibits differentiation of plasma cells and impairs the development of plasma cell growth factors in lymph node macrophages, thereby impeding the production of antibodies to influenza vaccination.^15^ Harris et al showed that the response to rotavirus vaccination correlated with the composition of the gut microbiome in infants in Pakistan.^16^ Reports suggest that the rotavirus vaccine works more effectively in infants born in developed countries than in those born in low income countries. To some extent, these differences have been attributed to the differences in gut microbiome composition.^17^

Herein, we investigated the relationship between the gut microbiome and rabies vaccine‐induced antibody‐mediated immune responses in mice. The generation of immune cells related to humoral immunity and the production of RABV‐specific antibodies were evaluated. We demonstrated that treatment with antibiotics resulted in decreased production of RABV‐specific antibodies after vaccination. We found that the abundances of *Clostridiales* and *Lachnospiraceae* in the mouse gut were positively associated with antibody production after RABV inoculation.

## 2 MATERIALS AND METHODS

### 2.1 Animals, viruses, cells, and antibodies

Female ICR mice were purchased from the laboratory animal center of Huazhong Agricultural University, Wuhan, China. The recombinant RABV strain LBNSE was derived from the Street Alabama Dufferin (SAD)‐L16, which is widely applied for the development of vaccine.^18^ LBNSE with two mutations in the G protein, N194K and R333E, was proliferated in BSR cells.^18^, ^19^ The rabies challenge virus strain‐11 (CVS‐11) was proliferated in BSR cells, too. The BSR cells, a cloned cell line come from BHK‐21 cells, were cultured at 37°C in Dulbecco's modified Eagle's medium (DMEM) (Mediatech, USA) containing 10% fetal bovine serum (FBS, Gibco) and antibiotics (penicillin‐streptomycin solution, 100×) (Beyotime, Wuhan, China). Fluorescein isothiocyanate (FITC)‐conjugated the antibodies against the RABV‐nucleoprotein (N) were obtained from FujiRab (Melvin, PA). Samples of dLNs were stained with monoclonal antibodies for flow cytometry, including FITC‐CD4 (BioLegend), APC‐CD185 (CXCR5) (BioLegend), PE‐CD279 (PD‐1) (BioLegend), FITC‐CD45R/B220 (BioLegend), Alexa Fluor 647‐GL7 (BioLegend), and PE‐CD95 (APO‐1/Fas) (eBioscience).

### 2.2 Broad‐spectrum antibiotics treatment, feces collection, and mouse inoculation

Female ICR mice, 3‐week‐old, (n = 10 per group) were provided with a cocktail of broad‐spectrum antibiotics (Abx, consisting of 1 g/L each of ampicillin, metronidazole, neomycin, and 0.5 g/L vancomycin, Sigma‐Aldrich) treatment for 28 days prior to immunization with rabies vaccines and continuously throughout the experiment to remove gut microbiome.^15^ The untreated female ICR mice, 3‐week‐old (n = 10), were administrated with sterile water as control. The feces collection of Abx‐treated mice and untreated mice were conducted weekly before immunization for fecal DNA extraction and quantification. The feces of Abx‐treated and untreated mice were collected on the same day with immunization for fecal DNA extraction as well as 16S rRNA gene sequencing. Groups of Abx‐treated and untreated ICR mice were inoculated with 10^7^ FFU formalin‐inactivated LBNSE (iLBNSE) (100 µL per mouse) by intramuscular (i.m.) route.

### 2.3 Single antibiotic treatment, feces collection, and mouse inoculation

Female ICR mice, 3‐week‐old (n = 10 per group), were solely provided with 1 g/L of ampicillin, 1 g/L of neomycin, 1 g/L of metronidazole, or 0.5 g/L of vancomycin (Sigma‐Aldrich) treatment for 28 days prior to inoculation and continuously throughout the experiment to remove gut microbiome.^15^ The untreated female ICR mice, 3‐week‐old (n = 10 per group), were fed with sterile water as control. The feces collection of vancomycin‐treated mice and untreated mice were conducted on the same day with vaccine inoculation for 16S rRNA gene sequencing. Groups of single antibiotic‐treated and untreated mice were inoculated with 10^7^ FFU iLBNSE (100 µL per mouse) by intramuscular (i.m.) route.

### 2.4 Feces collection, mouse inoculation, and group selection by virus‐neutralizing antibody titers

A total of 174 female ICR mice, 6‐8‐week‐old, were arranged for investigating the heterogenicity of virus‐neutralizing antibody (VNA) titers in response to rabies vaccination. The feces of 174 ICR mice were collected on the same day with vaccine inoculation and immediately stored at ‐80°C. The VNA titers were determined by FAVN test weekly for 3 weeks, and feces of mice with VNA titers higher than 7.79 IU/mL (H group, n = 26) and mice with VNA lower than 1.50 IU/mL (L group, n = 26) were selected for subsequent DNA extraction as well as 16S rRNA gene sequencing.

### 2.5 Fecal DNA quantification by quantitative real‐time PCR

Quantification of the fecal DNA was performed by quantitative real‐time PCR (qPCR) following the procedure described previously.^15^ Fecal genomic DNA extraction from stool samples was performed with TIANamp Stool DNA kit (TIANGEN, Beijing, China). The qPCR was performed for quantification of bacterial load using 2 µL of fecal DNA (<100 ng), 1 µL of the bacteria‐specific primer (10 µM) 27F (5′‐AGAGTTTGATCCTGGCTCAG‐3′), 1 µL of the universal primer 338R (10 µM) (5′‐CTGCTGCCTCCCGTAGGAGT‐3′), and 10 µL of AceQ Universal SYBR qPCR Master Mix (2×) (Vazyme, China) with a total of 20 µL qPCR reaction. The qPCR program began with 95°C for 8 min, followed by 40 cycles (95°C × 15 s, 60°C × 45 s). The primers 27F (5′‐AGAGTTTGATCCTGGCTCAG‐3′) as well as 1492R (5′‐GGTTACCTTGTTACGACTT‐3′) were used to generate the 1.5‐kb 16S rRNA amplicon from *Escherichia coli* that was subsequently cloned into pMD‐18T vector (TAKARA).^20^ The 10‐fold serial dilution of plasmid with known concentration was used as a standard for qPCR analysis to determine 16S rRNA copy numbers in fecal DNA samples.

### 2.6 DNA extraction, PCR amplification, and sequencing

All fecal samples were quickly frozen in liquid nitrogen for 15 min then stored at −80°C before DNA extraction. DNA extraction and 16S library preparation were conducted by Majorbio Bio‐Pharm Technology Co., Ltd. (Shanghai, China). Genomic DNA of fecal sample was extracted from 0.5 g feces by the application of the QIAmp Fast DNA Stool Mini Kit (Qiagen) in accordance with the manufacturer's protocols. The concentration and purity of extracted DNA were confirmed by NanoDrop 2000 UV‐VIS spectrophotometer (Thermo Scientific, USA). The quality of extracted DNA was ensured by agarose gel (2%). The hypervariable V3‐V4 regions of the bacterial 16S rRNA gene were PCR amplified by using the primer pairs 338F 5′‐ACTCCTACGGGAGGCAGCAG‐3′ and 806R 5′‐GGACTACHVGGGTWTCTAAT‐3′. The PCR amplification programs were conducted as follows: 95°C for 3 min (initial denaturation), 30 cycles (95°C × 30s (denaturation), 60°C × 30 s (annealing), 72°C × 45 s (elongation)) then 72°C for 10 min (final extension). The PCR was performed for 16S rRNA amplification using 1 µL of template DNA (10 ng), 0.8 µL of forward primer 338F (5 µM), 0.8 µL of reverse primer 806R (5 µM), 2 µL of dNTPs (2.5 mM), 0.4 µL of FastPfu polymerase, and 4 µL of FastPfu Buffer (5×) with a total of 20 µL PCR reaction. Amplicons were subsequently purified by the application of the AxyPrep DNA Gel Extraction Kit (Axygen Biosciences, USA). The purified amplicons were then sequenced by the MiSeq platform (Illumina, USA).

### 2.7 Sequencing analysis

Raw FASTA files from Illumina sequencing were analyzed at the website of Majorbio Cloud Platform (www.majorbio.com). The initial parameter setting for contrastive analysis was based on the study of Wei et al.^9^ Operational taxonomic units (OTUs) were clustered with ≥97% similarity cutoff. RDP classifier Bayesian algorithm was assigned to taxonomically compare the representative 16S rRNA gene sequences with 70% of confidence threshold against Silva database (silva 132/16s bacteria).

### 2.8 Measurement of VNA

VNA in blood samples derived from mice collected weekly from each mouse were measured. Quantitation was done by the application of the fluorescent antibody virus neutralization (FAVN) test that was described previously.^21^ In brief, 50 μL of blood samples and standard serum were threefold serially diluted and 100 μL of DMEM were added into 96‐well microplates. Each dilution of serum sample was repeated in quadruplicate. Each well was incorporated to 50 μL of the virulent RABV CVS‐11 strain containing 80–200 FFU and incubated at 37°C for 1 h. BSR cells (2 × 10^4^ cells per well) were incorporated to each well at the end of coculture of virus and serum. The plates of cells were incubated for 48 h at 37°C. Cells in the plates were fixed with 80% ice‐cold acetone for 30 min at ‐20°C, air dried, and washed with PBS for three times. FITC‐anti‐RABV N antibodies (1:500) were used to stain the cells and incubated at 37°C for 45 min followed with three times of wash with PBS and then analyzed using fluorescence microscope (Olympus IX51). VNA titers were calculated based on the result of the reference serum (WHO) and measured in international units per milliliter (IU/mL).

### 2.9 Flow cytometry

Flow cytometry was used to quantify follicular T helper (Tfh) cells and germinal center (GC) B cells in the draining lymph nodes (dLNs) that was described previously.^22^ Briefly, two dLNs on either side of the thigh from each mouse were obtained and pressed through nylon filter (40 μm). The single‐cell suspension was obtained and washed twice with PBS. The dLNs derived‐single‐cell suspensions (10^6^ cells) in PBS were blocked for 10 min with bovine serum albumin (BSA) (0.2%), and then stained with the corresponding antibodies for flow cytometry. After incubation at 4°C for 30 min, the cells were washed with PBS again and eventually resuspended with PBS for flow cytometry analysis. BD LSR‐II flow cytometer (BD Pharmingen) was applied to acquired data of samples, and FlowJo software (TreeStar, San Carlos, CA) was applied to analyzed data.

### 2.10 Enzyme‐linked immunosorbent assay

RABV‐specific IgM, IgG, and IgG isotypes in mice serum samples were measured using indirect ELISA as previously described.^23^ In brief, 500 ng per well of purified RABV virion proteins were used to coat wells of 96‐well plates overnight at 4°C. Coated plates were washed with PBS supplemented with 0.05% Tween 20 (PBS‐T), blocked with 5% skim milk for 2 h then washed three times again with PBS‐T. Serial dilutions of serum were aliquoted to wells and plates were incubated 2 h at 37°C. The plates were washed with PBS‐T and then incubated with horseradish peroxidase (HRP)‐conjugated Abs (IgG (1:2000), IgG1 (1:1500), IgG2a (1:1500), or IgG2b (1:2000)) (Boster, Wuhan, China) at 37°C for 1 h. The plates were then stained with 100 μL of the tetra‐methyl‐benzidine (TMB) substrate (Biotime Biotechnology, Shanghai, China) in the dark for 15–30 min followed with 50 μL of 2 M sulfuric acid. OD_450_ was measured using a SPARK10M multi‐function enzyme labeling instrument (TEACN, Austria).

### 2.11 ELISpot

ELISpot was performed to quantify the ASCs in the dLNs. Briefly, ELISpot plates (Dakewe, Shenzhen, P. R. China) were activated with 15 μL 35% ethanol then coated with 1 μg per well purified RABV virion proteins and incubated overnight at 4°C. Prior to cells being plated, the ELISpot plates were washed with sterile PBS for five times and then added with PRMI 1640 supplemented with 10% FBS and antibiotics (penicillin‐streptomycin solution, 100×) for blocking at 37°C for 2 h. Single‐cell suspensions from the dLNs, isolated 2 weeks post inoculation, were aliquoted to the appropriate wells and cultured at 37°C for 24‐36 h. The cells in the plates were washed away with ice water and incubated with Biotin‐mIgG antibody (Bethyl Laboratories, TX, USA) at 37°C for 2 h. The cells were washed with PBS for three times followed with a subsequent incubation with Streptavidin‐Alkaline Phosphatase (Mabtech, Stockholm, Sweden) at 37°C for 1 h. The plates were then stained with BCIP/NBT‐plus (Mabtech, Stockholm, Sweden) and the spot intensity was measured using an automated ELISpot reader.

### 2.12 Statistical analysis

The difference for RABV‐specific antibodies and numbers of immune cells were determined with Student's *t*‐test by the application of GraphPad Prism 8.0 (GraphPad Software, Inc., San Diego, CA). Unless otherwise noted, the data in this study are representative of the mean ± SEM. For all tests, ^*^ *P* < .05; ^**^ *P* < .01; ^***^ *P* < .001; ^****^P < .0001 represent significant differences among groups.

## 3 RESULTS

### 3.1 RABV‐specific antibody production is dependent on gut microbiome

**FIGURE 1:**
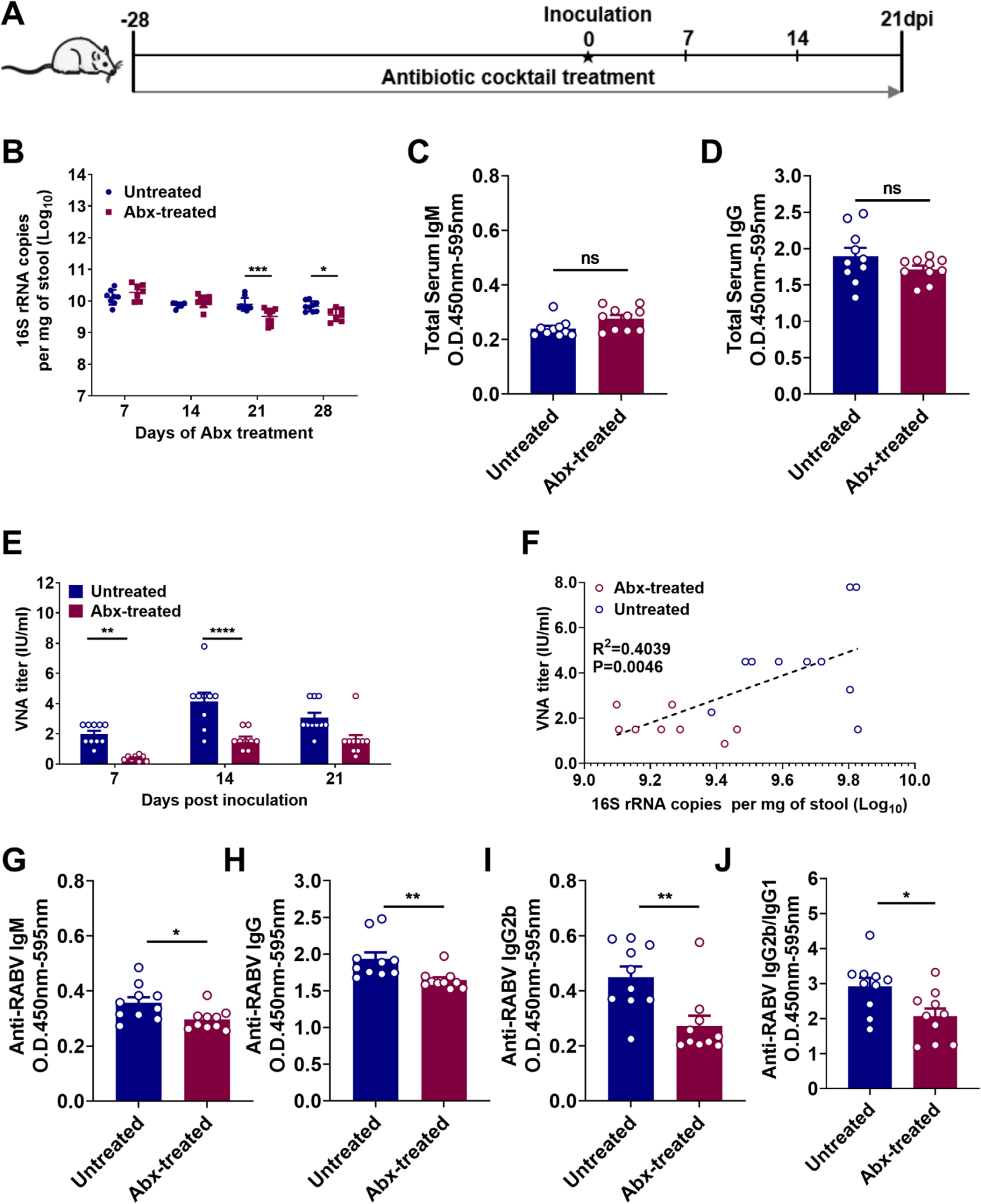
Gut microbiome is indispensable for optimal RABV‐specific antibody production. A, Schematic of treatment regimens. Female ICR mice, 3‐week‐old, (n = 10) were fed with a cocktail of antibiotic (Abx) treatment through drinking water for 28 days prior to vaccination and continuously throughout the experiment to deplete gut microbiome, or administrated with sterile water as negative control. The feces of Abx‐treated mice and untreated mice were collected weekly before vaccination for fecal DNA extraction and quantification. (Untreated mice, n = 10, Abx‐treated mice, n = 10). B, Bacteria in stool samples, collected at the indicated times, were quantified by qPCR. Error bars in the graphs represent standard error (^*^ *P* < 0.05; ^***^ *P* < 0.001; Student's *t*‐test). C,D, IgM and IgG in serum of mice was measured by ELISA after 28 days of Abx treatment. E, The Abx‐treated mice and untreated mice were i.m. inoculated with 10^7^ FFU rabies vaccine strain iLBNSE in the hind limbs. At indicated times, serum samples were collected for measurement of RABV‐specific VNA titers. F, Pair‐wise analysis of bacterial levels in stool vs RABV‐specific VNA titers at 14 days post inoculation. G‐J, Anti‐RABV IgM, IgG, IgG2b, and the IgG2b/IgG1 ratio were measured by indirect ELISA, and antibody levels in untreated mice were significantly higher than in Abx‐treated mice. Error bars in the graphs represent standard error (^*^ *P* < .05; ^**^ *P* < .01; Student's *t*‐test)

To evaluate the contribution of gut microbiome on humoral immunity post rabies vaccination, we depleted the gut microbiome by treating the mice with antibiotic (Abx) for 28 days prior to rabies vaccination and continuously throughout the experiment (Figure 1A). Mice in Abx‐treated group were orally administrated with a cocktail of ampicillin, metronidazole, neomycin, and vancomycin in their drinking water over 28 days pre‐inoculation to diminish Gram‐positive (vancomycin, ampicillin, and neomycin), Gram‐negative (neomycin), and anaerobic (metronidazole) bacteria in the gastrointestinal tract, and the treatment was continuously performed throughout the experiment. Mice in the control group were left untreated (untreated). The qPCR was used to quantify 16S rRNA in stool samples from all the mice. As shown in Figure 1B, 16S rRNA copies per gram of stool was significantly lower at 21 (*P* = .0006) and 28 (*P* = .0327) days in Abx‐treated mice verses untreated mice. To confirm that gut microbiome was depleted by Abx treatment, we examined the differences in the composition of the bacterial communities between Abx‐treated and untreated mice by the application of 16S rRNA gene sequencing, and the results showed that the Abx treatment caused a significant reduction in the composition of gut microbiome (Figure S1). The levels of total IgM (Figure 1C) and IgG (Figure 1D) in the serum of untreated mice were not significantly different from those in Abx‐treated mice, demonstrating these antibiotics do not affect the basal level of humoral immunity in mice.

To assess the role of the gut microbiome in modulating humoral immune responses induced by rabies vaccination, we evaluated the production of RABV‐specific antibody in mice over 3 weeks after inoculation with 10^7^ FFU rabies vaccine strain iLBNSE (i.m.). At the indicated time points, 16S rRNA in stool samples from all the mice were collected and quantified by qPCR (Figure S2). Meantime, RABV‐specific VNA titers were quantified by FAVN test.^21^ Abx‐treated mice had two‐ to fourfold lower VNA titers than untreated mice (*P* = .008 at 21 days and *P* < .0001 at 28 days; Figure 1E). Using pair‐wise analysis, it was showed that there existed a significant correlation between the number of bacteria in stool samples and RABV‐specific VNA titers in corresponding serum samples in the Abx‐treated mice (*P* = .0046; Figure 1F). We also compared levels of anti‐RABV IgG, IgM, and IgG isotypes in Abx‐treated and untreated mice using indirect ELISA^23^ at 14 days post vaccination. The levels of anti‐RABV IgM (*P* = .0184; Figure 1G), IgG (*P* = .0087; Figure 1H), IgG2b (*P* = .0037; Figure 1I), and the ratio of IgG2b/IgG1 (*P* = .0171; Figure 1J) in Abx‐treated mice were significantly lower than those in untreated mice. These results indicate that depletion of gut microbiome impairs RABV‐specific antibody production post vaccination.

### 3.2 Gut microbiome facilitates the recruitment of Tfh, GC B cells, and ASCs post rabies vaccination

**FIGURE 2:**
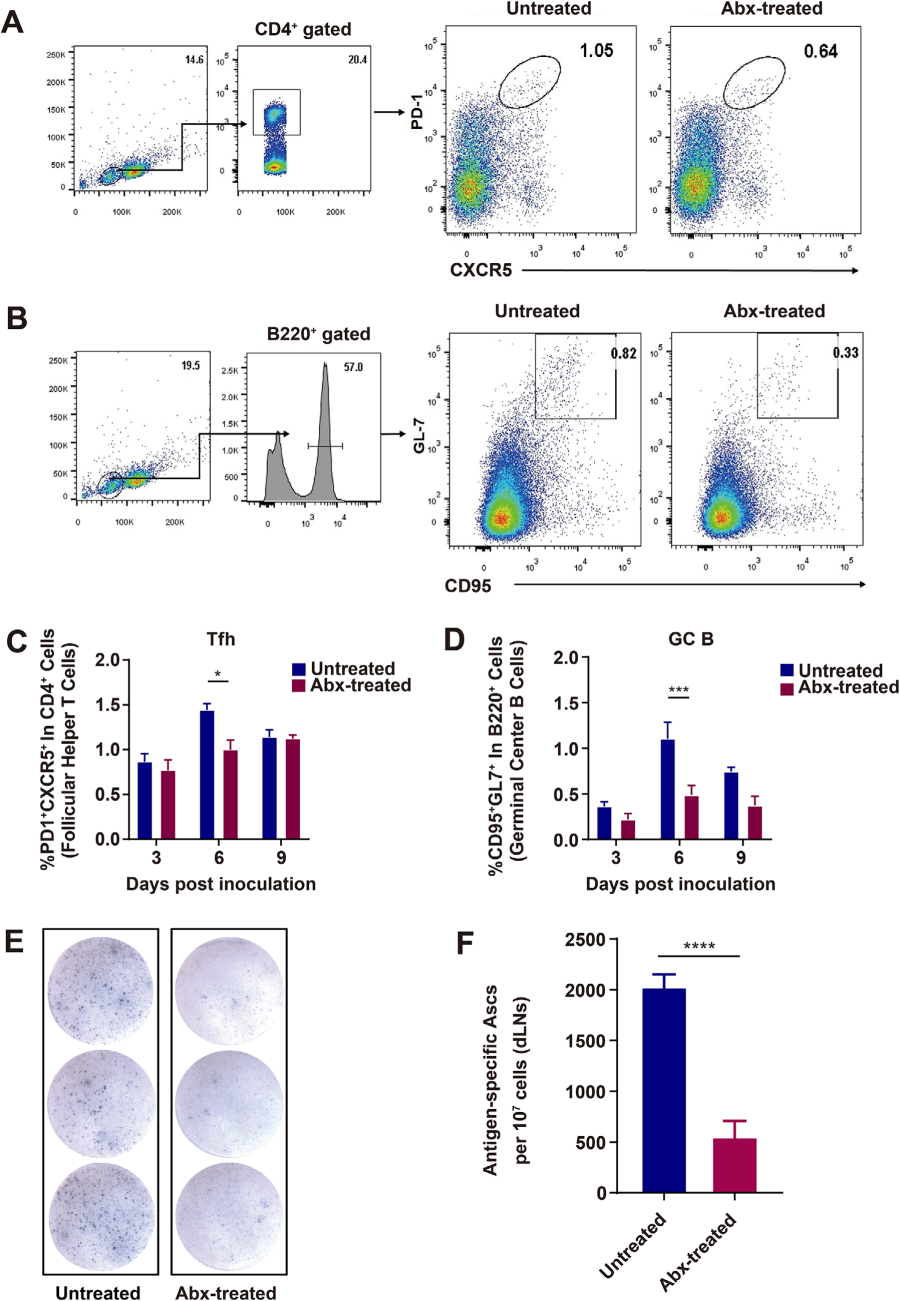
Recruitment of Tfh, GC B cells, and ASCs in Abx‐treated and untreated mice immunized with rabies vaccine. A‐D, Tfh and GC B cells in the draining LNs (dLNs) post rabies vaccination. Abx‐treated and untreated mice were inoculated i.m. with 10^7^ FFU iLBNSE. dLNs were harvested, and single‐cell suspensions of the dLNs were stained with Tfh and GC B cell markers, and analyzed by flow cytometry (Untreated mice, n = 5, Abx‐treated mice, n = 5). A, Representative gating strategy for identification of Tfh cells. B, From the dLNs, the percentage of PD1^+^ CXCR5^+^ Tfh cells in CD4^+^ B cells. Error bars in the graphs represent standard error (^*^ *P* < .05; Student's *t*‐test). C, Representative gating strategy for identifying GC B cells. D, From the dLNs, percentage of GL7^+^ CD95^+^ GC B cells in B220^+^ B cells. Error bars in the graphs represent standard error (^***^ *P* < .001; Student's *t*‐test). E,F, Quantification of ASCs in the draining LNs of Abx‐treated and untreated mice post immunization. Single‐cell suspensions of the dLNs were added into ELISpot plates coated with purified RABV, and subsequently incubated with Biotin‐mIgG Ab and Streptavidin‐Alkaline Phosphatase, signal was detected with BCIP/NBT‐plus (Untreated mice, n = 5, Abx‐treated mice, n = 5). E, Representative ELISpot images of ASCs from the draining LNs. F, Graph of ELISpot results. Error bars represent standard error ^(^ ^****^P < .0001; Student's *t*‐test)

Follicular helper T cells (Tfh) are crucial for the formation and maintenance of germinal centers (GC) where B cells differentiate and become affinity matured.^24^, ^25^, ^26^ To investigate whether the gut microbiome facilitates the recruitment of Tfh cells in vivo, Abx‐treated and untreated mice were inoculated with 10^7^ FFU iLBNSE (i.m.), and Tfh cells (CD4^+^ PD1^+^ CXCR5^+^) of the dLNs were quantified via flow cytometry at 3, 6, and 9 days post inoculation (dpi). Representative flow cytometric data on CD4^+^ cells and Tfh cells at 6 dpi are shown in Figure 2A. A higher abundance of Tfh cells were observed in the dLNs of untreated mice than in Abx‐treated mice at 6 dpi (*P* = .0006; Figure 2C). These data demonstrate that gut microbiome facilitates the generation and recruitment of Tfh cells in vivo.

GC B cells (B220^+^ CD95^+^ GL7^+^) in the dLNs were also quantified at 3, 6, and 9 dpi. Representative flow cytometric data on B220^+^ cells and GC B cells at 6 dpi are shown in Figure 2B. A higher abundance of GC B cells was discovered in the dLNs of untreated mice than in Abx‐treated mice at 6 dpi (*P* = .0118; Figure 2D). These data suggest that gut microbiome helps promote the generation of GC B cells in the dLNs of mice.

Germinal centers are the location for B cell clonal proliferation and antigen affinity‐based tendency, which cultivates opportunities for ASC differentiation.^27^ To further investigate whether GC B cells in the dLNs contribute to the generation of ASCs, ELISpot was performed to quantify the number of ASCs. As expected, significantly more ASCs were induced in untreated mice than in Abx‐treated mice (*P* < .0001; Figure 2E,F). The data mentioned above demonstrate that gut microbiome facilitates the generation of Tfh, GC B cells, and ASCs post rabies vaccination.

### 3.3 Gut microbiome helps to recall the secondary B cells responses

**FIGURE 3:**
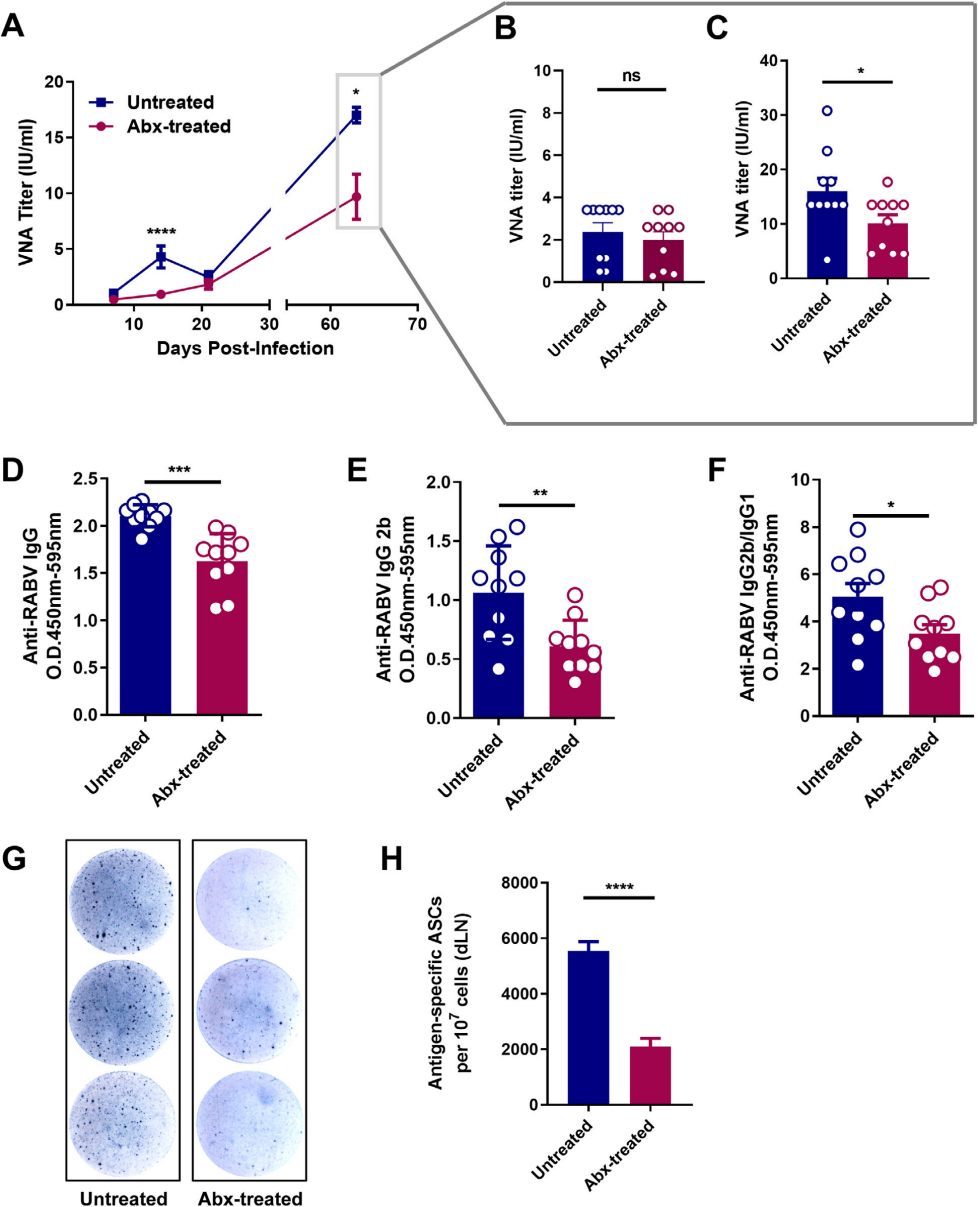
Impact of gut microbiome on the secondary immune responses. A‐C, RABV inoculated mice were boosted with 10^7^ FFU iLBNSE 56 days after the primary inoculation (Untreated mice, n = 10, Abx‐treated mice, n = 10). A, Serum VNA titers were measured by FAVN test. Error bars in the graphs represent standard error (^*^ *P* < .05; ^****^ *P* < .0001; Student's *t*‐test). B, Serum VNA titers before the boost and (C) 14 days after the boost. Error bars in the graphs represent standard error (^*^ *P* < .05; Student's *t*‐test). D‐F, Anti‐RABV IgG, IgG2b, and the IgG2b/IgG1 ratio were measured by indirect ELISA, and antibody levels in untreated mice were significantly higher than in Abx‐treated mice. Error bars in the graphs represent standard error (^*^ *P* < .05; ^**^ *P* < .01; ^***^ *P* < .001; Student's *t*‐test). G, Representative ELISpot images of ASCs from the dLNs of Abx‐treated and untreated mice (Untreated mice, n = 5, Abx‐treated mice, n = 5). H, ASCs in the dLNs were quantified. Error bars represent standard error (^****^ *P* < .0001; Student's *t* test)

To further demonstrate the effect of gut microbiome on the secondary immune responses to rabies vaccine, inoculated mice were boosted with 10^7^ FFU iLBNSE after 56 days post the primary inoculation. At 14 days after the boost, Abx‐treated mice exerted significantly lower VNA titers than untreated mice (*P* = .0455; Figure 3A‐C). Similarly, the levels of anti‐RABV IgG (*P* = .0001; Figure 3D), IgG2b (*P* = .0052; Figure 3E), and the IgG2b/IgG1 ratio (*P* = .0323; Figure 3F) in Abx‐treated mice were significantly lower than that in untreated mice.

To determine whether gut microbiome affects the generation of ASCs derived from memory B cells, the number of ASCs in the dLNs from Abx‐treated and untreated mice were determined 14 days after the boost. As expected, dramatically reduced ASCs were observed in Abx‐treated mice compared with those in untreated mice (*P* < .0001; Figure 3G,H). Taken together, these results suggest that gut microbiome is important for maintaining secondary immune responses.

### 3.4 Vancomycin treatment impairs bacterial community composition and humoral immunity induced by rabies vaccines

**FIGURE 4:**
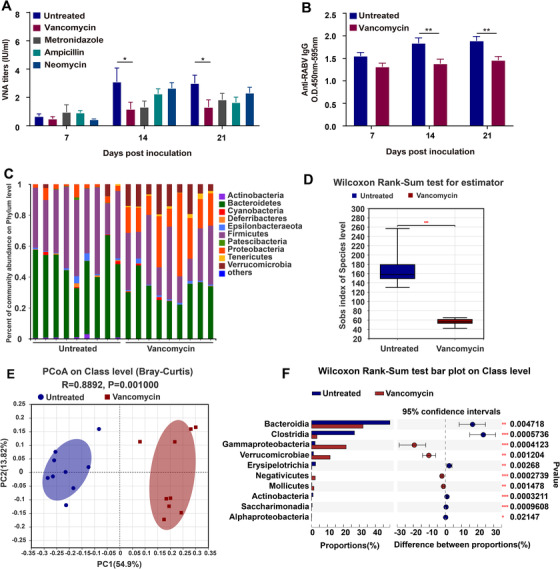
Vancomycin treatment impairs humoral immunity induced by rabies vaccine and bacterial community composition. A,B, Mice were treated with a single antibiotic for 28 days prior to and during rabies vaccination (Untreated mice, n = 9, Vancomycin‐treated mice, n = 9, Metronidazole‐treated mice, n = 9, Ampicillin‐treated mice, n = 9, Neomycin‐treated mice, n = 9). Antibody titers were determined by (A) FAVN test or (B) ELISA. Error bars in the graphs represent standard error (^*^ *P* < .05; ^**^ *P* < .01; Student's *t*‐test). C‐F, Composition of microbiota in untreated or vancomycin‐treated mice (Untreated mice, n = 9, Vancomycin‐treated mice, n = 9). C, Relative abundance of specific bacterial phyla in untreated and vancomycin‐treated mice. D,E, Alpha diversity and beta diversity of the bacterial community. Sobs index based on Wilcoxon Rank‐Sum Test at the OTU level and principal coordinate analyses (PCoA) based on Bray‐Curtis dissimilarity illustrates the composition of stool microbiota in untreated and vancomycin‐treated mice

For a more in depth look at the bacteria affecting RABV‐specific antibody production, we treated mice with one of each of the antibiotics from the cocktail for 28 days before rabies vaccination. We found that VNA (*P* = .0128 at 14 days and *P* = .0421 at 21 days) and total IgG (*P* = .0042 at 14 days and *P* = .0071 at 21 days) titers in vancomycin‐treated mice were significantly lower throughout the testing period than in mice treated with the other antibiotics as well as in untreated mice (Figure 4A,B).

By the application of 16S rRNA gene sequencing, we examined the differences in the composition of the bacterial communities in vancomycin‐treated and untreated mice. The raw sequencing data were spliced, filtered, and the remaining 33 739 sequences were clustered into 964 bacterial OTUs obtained from 18 mouse stool samples. We found differences in the relative abundance of several bacterial taxa between the two groups (Figure 4C‐F). The abundance of Bateroidetes and Firmicutes, the two most abundant phyla in the microbiome of untreated mice, (Figure 4C), were significantly reduced in vancomycin‐treated mice. Additionally, appearance of other augments was observed, and the most notable were Verrucomicrobia and Proteobacteria, respectively. The sobs index of OTU level in vancomycin‐treated mice exhibited significant decrease (*P* < .001) in the alpha diversity of the bacterial community compared with that in untreated mice. (Figure 4D). To compare overall diversity in the microbial communities, we used the Bray‐Curtis algorithm, the results were visualized using principal coordinate analyses (PCoA) (*R* = .8892, *P* = .001000) (Figure 4E). At the class level, six taxa were decreased in vancomycin‐treated mice, including Bacteroidia, Clostridia, Erysipelotrichia, Actinobacteria, Saccharimonadia, and Alphaproteobacteria, while Gammaproteobacteria, Verrucomicrobiae, Negativicutes, and Mollicutes were significantly increased (Figure 4F).

**FIGURE 5:**
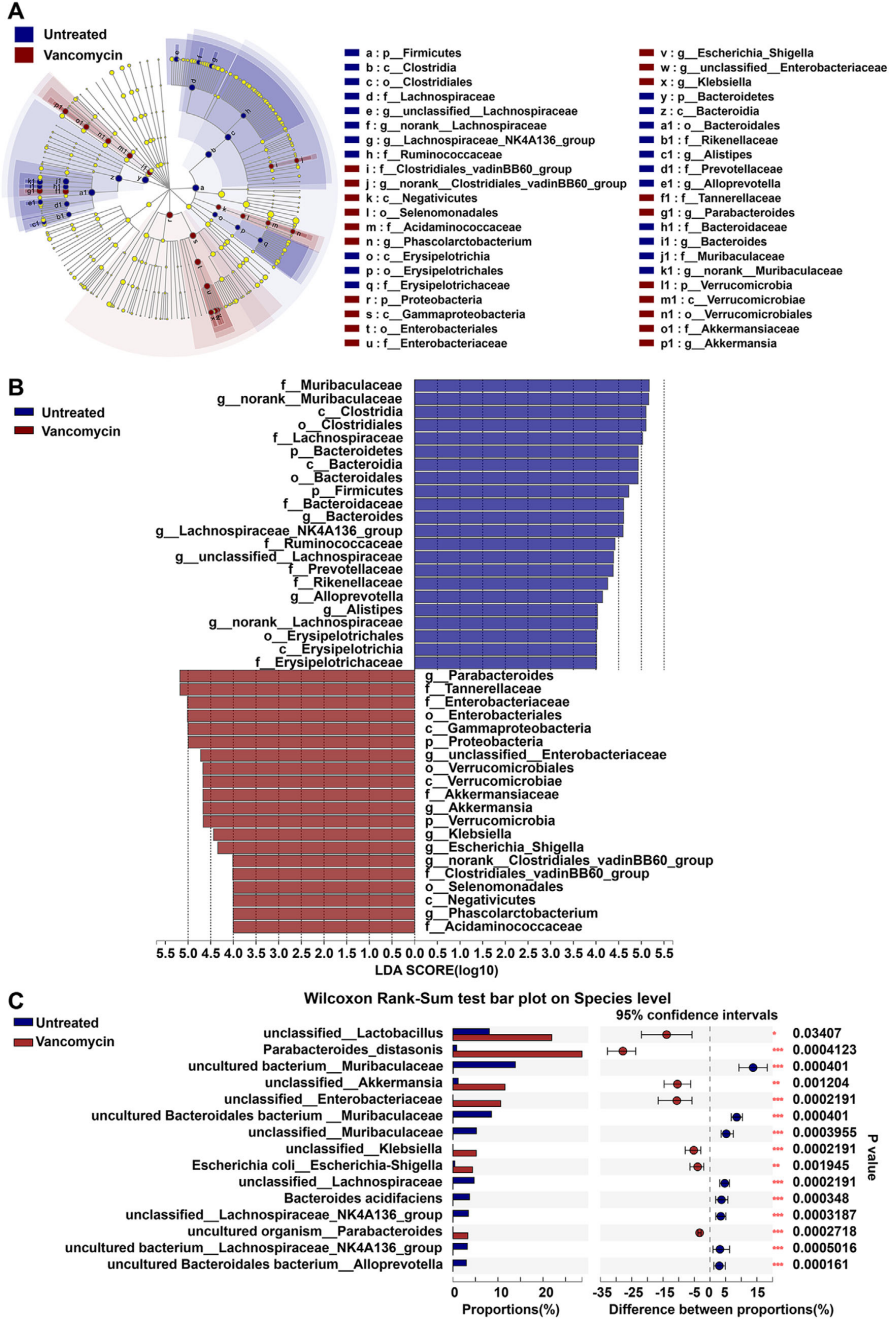
Meta‐analysis of specific microbiome associated with vancomycin treatment. A,B, LEfSe analysis revealed that the relative abundance of 42 taxa of bacteria was significantly different between the vancomycin‐treated and untreated mice at the different taxonomic levels. (Untreated mice, n = 9, Vancomycin‐treated mice, n = 9) (LDA > 4, *P* < .05). C, The Wilcoxon Rank‐Sum Test demonstrates the relative abundance of bacterial taxa at the species level was significantly different between vancomycin‐treated and untreated mice. (^*^ *P* < .05; ^**^ *P* < .01)

The composition of the microbiome of the vancomycin‐treated and untreated mice was then subjected to LEfSe analysis.^28^ Significant taxa differences were found between two groups at the phyla level (Figure 5A,B). A total of 42 differential bacterial taxa were identified by using LEfSe analysis (LDA score > 4.0, *P* < .05), including 22 increased abundance and 20 decreased abundance. In the vancomycin‐treated group, there were enhanced abundance in genera such as *Parabacteroides*, unclassified *Enterobacteriaceae, Akkermansia, Klebsiella, Escherichia_Shigella*, norank *Clostridiales_vadinBB60_group, Phascolarctobacterium*, and diminished abundance in genera such as norank *Muribaculaceae, Bacteroides, Lachnospiraceae_NK4A136_group*, unclassified *Lachnospiraceae, Alloprevotella, Alistipes*, and norank *Lachnospiraceae*. The top 15 different species level were shown in Figure 5C. Seven bacteria (unclassified *Lactobacillus, Parabacteroides distasonis*, unclassified *Akkermansia*, unclassified *Enterobacteriaceae*, unclassified *Klebsiella, Escherichia coli*, and unclassified *Parabacteroides*) dominated in vancomycin‐treated mice, and eight bacteria (uncultured bacterium *Muribaculaceae*, uncultured *Bacteroidales_bacterium Muribaculaceae*, unclassified *Muribaculaceae*, unclassified *Lachnospiraceae, Bacteroides_acidifaciens*, unclassified *Lachnospiraceae_NK4A136_group*, uncultured bacterium *Lachnospiraceae_NK4A136_group*, unclassified *Muribaculaceae*, and uncultured Bacteroidales bacterium *Alloprevotella*) showed a remarkable decrease in vancomycin‐treated mice compared with untreated mice (Figure 5C).

### 3.5 Gut microbiome composition is associated with RABV‐specific antibody production in the natural population of mice

**FIGURE 6:**
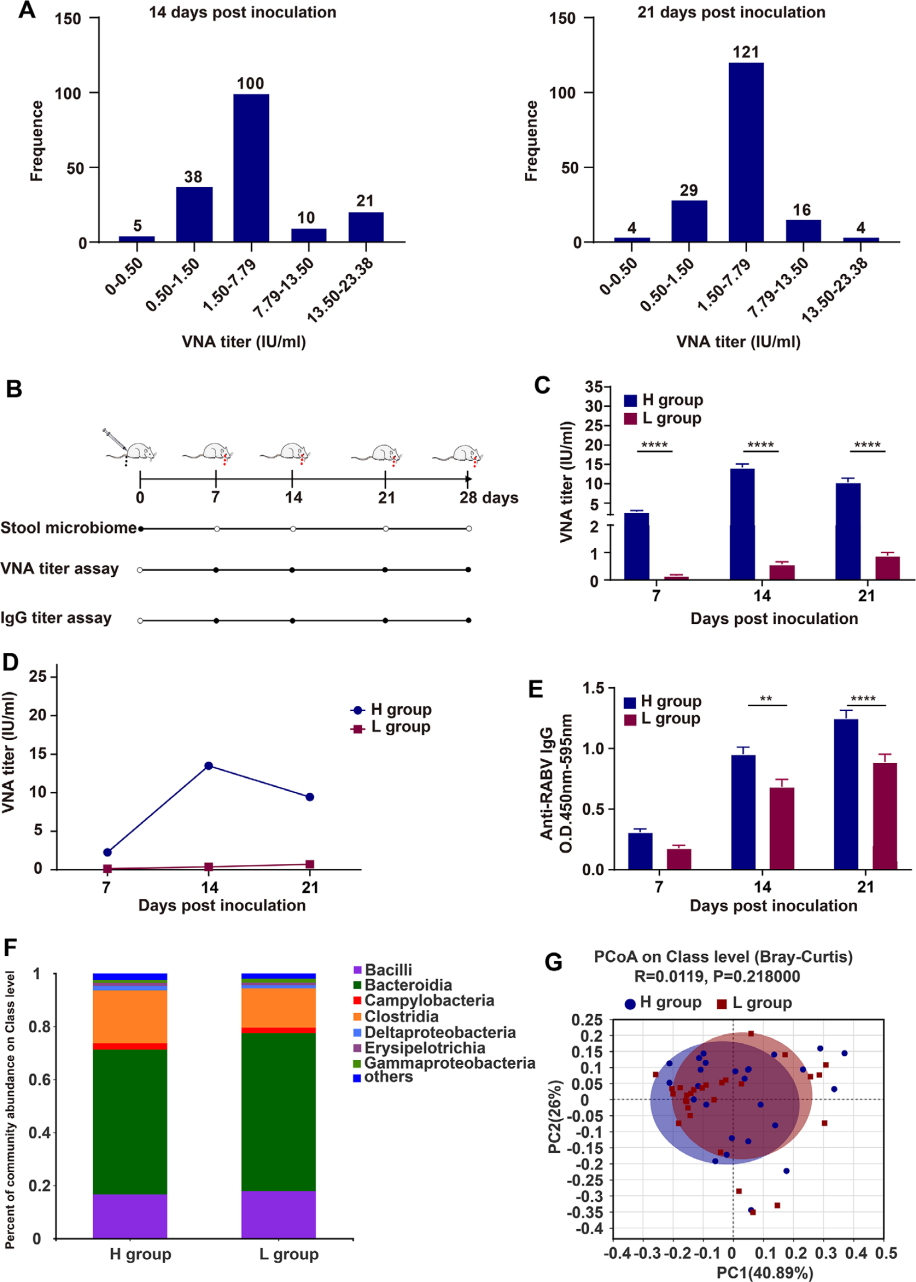
Gut microbiome composition is associated with anti‐RABV antibody production in the natural population of mice. A, Serum VNA titers in the natural population of mice immunized with rabies vaccines. Total 174 female ICR mice aged 6‐8 weeks were intramuscularly (i.m.) inoculated with 10^7^ FFU iLBNSE in gastrocnemius muscle of the right hind limb, and VNA titers in the serum were measured by FAVN test at 14‐ and 21‐days post vaccination, respectively. B, Schematic of 16S rRNA screening. ICR mice were i.m. inoculated with 10^7^ FFU iLBNSE. Then samples were collected and analyzed at regular intervals (black circles). C‐E, VNA and total IgG titers in VNA titer high (H) and low (L) group were determined by FAVN test and ELISA (H group, n = 26, L group, n = 26). The arithmetic mean of VNA titers (C), geometric mean of VNA titers (D), and total IgG level (E) was shown. Error bars represent standard error (^**^ *P* < .01; ^****^ *P* < .0001; Student's *t*‐test). F, Composition and relative abundance of gut microbiome at the class level. G, Principal coordinate analyses (PCoA) based on Bray‐Curtis illustrates the similarity of the fecal microbiota in H and L group

By using Abx‐treatment, we have identified that several bacteria are tightly associated with the humoral immunity induced by rabies vaccines. To further confirm the impact of gut microbiome on rabies vaccination in the natural population of mice without Abx‐treatment, 174 female ICR mice, aged 6‐8 weeks, were inoculated with 10^7^ FFU of iLBNSE, and blood samples were collected at weekly intervals to determine VNA titers by FAVN test. We found conspicuous differences in VNA titers among the mice at 14‐ and 21‐days post inoculation (Figure 6A). The highest VNA titer was 23.38 IU/mL and lowest was 0.17 IU/mL, demonstrating the diversity of humoral immunity to rabies vaccines.

Of the 174 mice inoculated with iLBNSE, 16S rRNA gene sequencing was performed on fecal samples from 26 mice with VNA titers higher than 7.79 IU/mL (H group) and 26 mice with VNA lower than 1.50 IU/mL (L group). Average VNA titers in the H group were 5‐ to 10‐fold higher than in the L group (*P* < .0001 at 7, 14, and 21 days; Figure 6C,D); the levels of RABV‐specific IgG in the H group were significantly higher than those in the L group as well (*P* = .0014 at 14 days and *P* < .0001 at 21 days) (Figure 6E). We found a significantly positive correlation between VNA titers and the level of Clostridia (*P* = .04) and a negative correlation with the level of Gammaproteobacteria (*P* = .06) at class level (Figure 6F). This trend was similar with that we observed in vancomycin‐treated and untreated mice. Notwithstanding, there was barely any difference in microbial beta‐diversity between the H and L group mice by using ANOSIM analysis (*R* = 0.0119, *P* = .218000; Figure 6G).

### 3.6 Meta‐analysis of specific microbiota associated with humoral immunity post rabies vaccination

**FIGURE 7:**
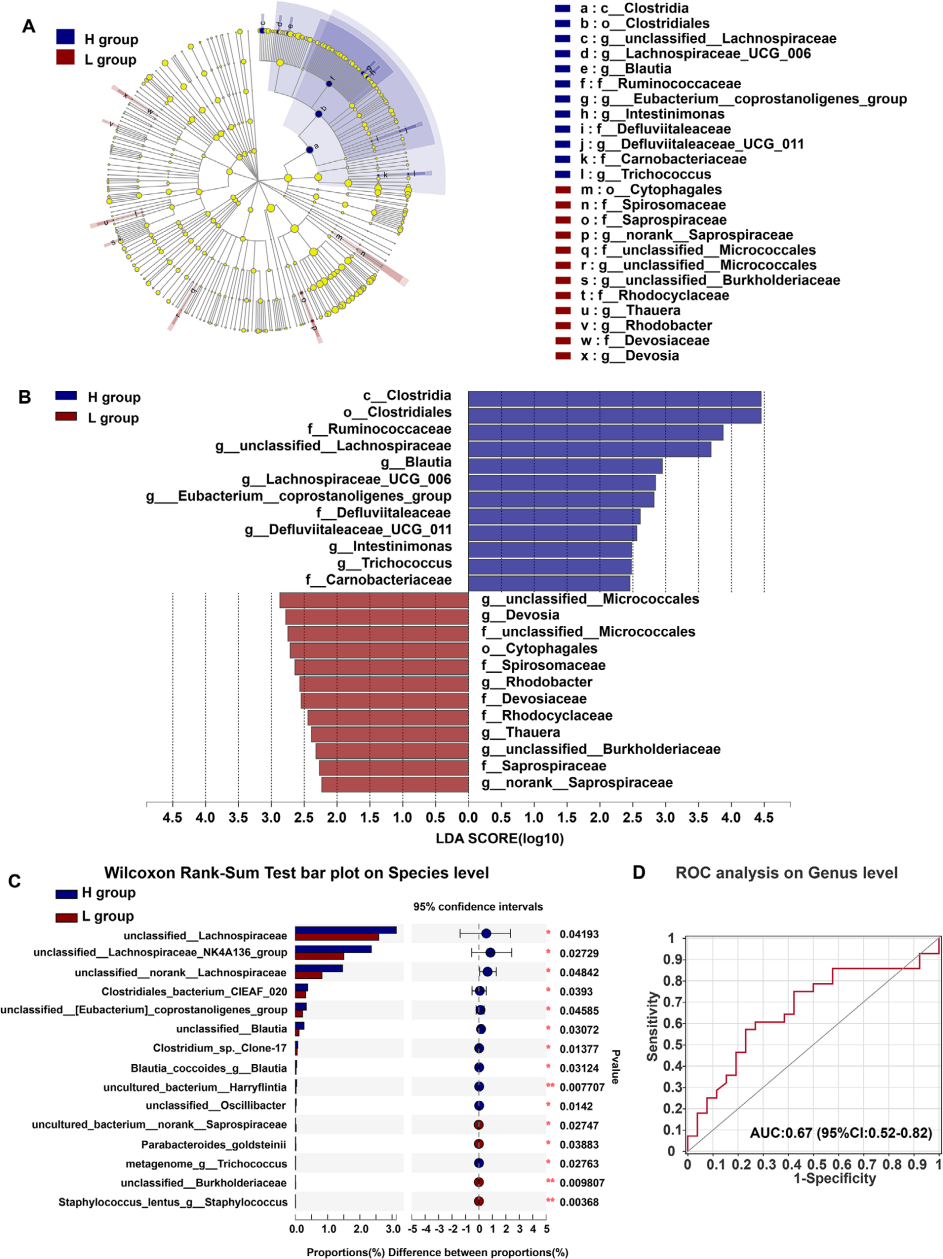
Meta‐analysis of specific microbiome associated with humoral immunity post rabies vaccination. A,B, LEfSe analysis revealed that the relative abundance of 23 taxa of bacteria was significantly different between the H and L group at the different taxonomic levels. (H group, n = 26, L group, n = 26). (LDA > 2, *P* < .05). C, The Wilcoxon Rank‐Sum Test demonstrates the relative abundance of bacterial taxa at the species level was significantly different between H and L group. (^*^ *P* < .05; ^**^ *P* < .01). D, Classification performance of multivariable logistic regression model using relative abundance of H groups‐associated genera was assessed by area under receiving operating characteristics curve

The composition of the microbiome of the H group and L group was subjected to LEfSe analysis.^28^ Significant differences could be observed between two groups at the family level (Figure 7A,B). Total of 23 differential bacterial taxa were identified by using LEfSe analysis (LDA score > 2.0, *P* < .05), including 11 increased abundance and 12 decreased abundance. In the H group, there were enhanced abundance in genera such as of unclassified *Lachnospiraceae, Blautia, Lachnospiraceae_UCG_006, Eubacterium_coprostanoligenes_group, Defluviitaleaceae_UCG_011, Intestinimonas*, and *Trichococcus*, and diminished abundance of unclassified *Micrococcales, Devosia, Rhodocyclaceae, Thauera*, unclassified *Burkholderiaceae*, and norank *Saprospiraceae*. The differences in abundance between H and L group at the species level were calculated using the Wilcoxon Rank‐Sum Test. Figure 7C listed the top 15 different species, and most of them were from *Clostridiales* and *Lachnospiraceae*.

We next assessed the value of using particular gut microbiota as a diagnostic for high antibody production. Using the combination of species, unclassified *Lachnospiraceae, Blautia, Lachnospiraceae_UCG_006, Eubacterium_coprostanoligenes_group, Trichococcus*, and *Defluviitaleaceae_UCG_011*, we generated an area under receiving operating characteristics curve (AUROC) of 0.66 (95% CI: 0.51 to 0.81; Figure 7D). Together, these data demonstrate that bacterial abundance, particularly of *Clostridiales* and *Lachnospiraceae*, is positively correlated with antibody levels induced by rabies vaccination.

## 4 DISCUSSION

Antibiotic‐induced microbiome depletion has been widely implicated in immune conditions that impair antibody responses to influenza^1^, ^15^, ^29^ and rotavirus vaccination^16^ as well as the mucosal adjuvant activity of cholera toxin.^30^ Numerous studies have reported that the microbiome or its metabolites play a role in B cell proliferation and differentiation by triggering activation of multiple pathways or ligands, such as B cell receptor (BCR), CD40, and Toll‐like receptors (TLRs), thereby increasing B cell survival and governing innate and adaptive immunity.^31^, ^32^ By the application of both Abx‐treated and germ‐free mice model, it was demonstrated that gut microbiome contributed to the development of IgG and IgM after influenza vaccination.^15^, ^29^ Our findings in ICR mice treated with antibiotics were consistent with these results; Abx‐treated mice showed depressed levels of RABV‐specific IgM, IgG, and VNA titers. In this study, we addressed the impact of the gut microbiome in humoral immunity in mice post rabies vaccination. Our data showed that a depleted microbiome resulted in depressed recruitment of a specialized subset of CD4 ^+^ T cells, Tfh, and GC B cells. The association of Tfh and GC B cells subsequently influenced the generation of specific ASCs. Additionally, we also found the microbiome impacted the secondary B cell responses after rabies vaccination.

The composition of the gut microbiome has been shown to influence humoral immune responses.^33^, ^34^ Elevated levels of Actinobacteria were positively correlated with humoral immune responses to hepatitis B oral vaccination in infants, while higher levels of Enterobacter were negatively correlated.^35^ In our study, it was revealed that Actinobacteria showed decreased generation at class level and *E. coli* showed dominant abundance at species level after vancomycin treatment, and Gammaproteobacteria abundance was negative in accordance with humoral immunity post rabies vaccination in mice, as expected. Other researchers have reported that elevated levels of *Clostridium cluster* XI and Proteobacteria are positively correlated with the Rotavirus vaccine response in infants.^16^ Our data were partially coincident with these results. Comparing mice with high levels of rabies‐specific VNA titers and mice with low levels revealed a significant positive correlation between VNA titer and abundance of Clostridia (*P* = .04), but a negative correlation with Gammaproteobacteria abundance (*P* = .06) at class level. Additionally, Harris et al^36^ showed a significant positive correlation between the RVV response and the Bacilli phylum, particularly *Streptococcus bovis* and a significant negative correlation between the RVV response and the Bacteroidetes phlyum, especially some *Bacteroides* and *Prevotella* genera. In our study, we observed that vancomycin‐treated mice had lower RABV‐specific VNA titers than did mice treated with the other single antibiotics and untreated mice. 16S rRNA gene sequencing revealed vancomycin‐treated mice had decreased populations of Bacteroidia, Clostridia, Erysipelotrichia, Actinobacteria, Saccharimonadia, and Alphaproteobacteria but abundant Gammaproteobacteria, Verrucomicrobiae, Negativicutes, and Mollicutes. By administrating mice with vancomycin, we found a notable decrease in the population of *Clostridiales* and *Lachnospiraceae*, and a coincident decrease in the production of RABV‐neutralizing antibodies. In summary, our results demonstrated that gut microbiome impacted humoral immunity post rabies vaccination.

*Clostridiales* are Gram‐positive bacteria belonging to the class Clostrida in the phylum Firmicutes. Accumulating evidences show that the *Clostridiales* play a critical role in the modulation of host immune function. Vancomycin is typically used as inhibitor of *Clostridium* species in vivo.^37^ In mice, oral administration of a mixture of Clusters IV and XIVa *Clostridum* strains promoted the accumulation of CD4^+^ T regulatory cells, thereby playing an important role in anti‐inflammation. The treated mice also showed a higher resistance to colitis and allergy than untreated.^38^ A mixture of Clostrida strains from the human microbiota had similar effects on mice models of colitis and allergic diarrhea.^39^ It was reported that in pediatric stem cell transplant patients, the development of graft‐versus‐host‐disease (GVHD) was associated with antibiotic depletion of anti‐inflammatory Clostridia, these patients also had a relative increase in levels of pro‐inflammatory gram‐negative bacteria (Enterobacteriaceae).^40^ Our study demonstrated a positive correlation between Clostrida abundance, particularly *Clostridiales* and *Lachnospiraceae*, and the production of RABV‐specific antibodies post vaccination.

Recently, germ‐free (GF) mice are introduced to explore the function of the gut microbiome in adaptive immune responses.^4^, ^41^, ^42^ GF mice have a small number of intraepithelial lymphocytes and have difficulty in balancing helper T‐cell subsets, resulting in a depressed immune response. Although GF mice are an optimal model for studying the relationship between immunity and the gut microbiome, they need strictly sterile feeding environment and high breeding expenses. Thus, prebiotic and probiotic treatments are often used in gut microbiological research; the combination of treatment with inulin had a positive effect on the growth of butyrate‐producing *Clostridium cluster* XIVa and *Blautia*, and correlated with antitumor activity and a decrease of body mass index.^43,44^

It is generally accepted that microbiome or their metabolites exert an influence on vaccine immune responses. For example, short‐chain fatty acids (SCFAs) and flagellin are potent immune activators. Administration of SCFAs to mice facilitated the activity of mucosal adjuvant cholera toxin by promoting the production of BAFF and ALDH1a2 from dendritic cells (DCs) and subsequently B cell Ab production.^45^ Flagellin was recognized by the pattern recognition receptor TLR5, activating downstream signaling pathways that resulted in elevated levels of mucosal and systemic antibodies and activation of cellular immune responses.^46^, ^47^ Our previous study also showed that a recombinant RABV expressing flagellin contributed to VNA production and recruitment of DCs.^48^

## 5 CONCLUSIONS

Our data demonstrate the connection between the gut microbiome and humoral immunity in mice after rabies vaccination, which provide insights into the effectiveness of vaccines in general and rabies vaccines in particular. Gut microbiome is source of natural adjuvants that contributes to activating a multitude of pathways that control innate and adaptive immunity.^32^ Further investigation of the specific mechanisms by which microorganisms or their metabolites modulate the immune effect of vaccines will contribute to the development of novel adjuvants and possibly the personalized design of vaccines.

## AUTHOR CONTRIBUTIONS

Y. Z., M. Z., and Q. W. designed the study and conducted the work. Y. Z. and Q. W. wrote the manuscript. Y. Z., Q. W., J. P., C. W., Z. L., L. L., B. C., B. S., and F. H. performed the experiments. Z. F. and L. Z. analyzed the data. All authors contributed to manuscript revision and approved the submitted version.

## AVAILABILITY OF DATA AND MATERIALS

The raw 16S sequencing data reported in this study is deposited at NCBI Sequence Read Archive (SRA) under accession number PRJNA611837.

## ETHICS APPROVAL AND CONSENT TO PARTICIPATE

All animal experiments performed in this study were strictly following the recommendations in the Guide for the Care and Use of Laboratory Animals of the Ministry of Science and Technology of the People's Republic of China. All animal experiments were approved by the Scientific Ethics Committee of Huazhong Agricultural University (permit number HZAUMO‐2018‐048).

## CONFLICT OF INTEREST

The authors declare that they have no conflict of interest.

## Supporting information

Supporting Information.
