# Supplementary material for: Composition of the murine gut microbiome impacts humoral immunity induced by rabies vaccines
Source: Clin Transl Med. 2020 Aug 22;10(4):e161. doi: 10.1002/ctm2.161 (PMC7443138; doi:10.1002/ctm2.161)
Supplement: Supplementary file 1 — Supporting Information. [file CTM2-10-e161-s001.docx]

Supporting Information

**Composition of the murine gut microbiome** **impacts humoral immunity induced by rabies vaccines**

Yachun Zhang^a,b†^, Qiong Wu^a,b†^, Ming Zhou^a,b^, Zhaochen Luo^a,b^, Lei Lv^a,b^, Jie Pei^a,b^, Caiqian Wang^a,b^, Benjie Chai^a,b^, Baokun Sui^a,b^, Fei Huang^a,b^, Zhen F. Fu^a,b^, Ling Zhao^a,b*^

*^a^ State Key Laboratory of Agricultural Microbiology, Huazhong Agricultural University, Wuhan, 430070, China*

*^b^ Key Laboratory of Preventive Veterinary Medicine of Hubei Province, College of Veterinary Medicine, Huazhong Agricultural University, Wuhan, 430070, China*

^†^ These authors contributed equally to this work.

*To whom correspondence should be addressed:

Ling Zhao: State Key Laboratory of Agricultural Microbiology, Huazhong Agricultural University, Wuhan, 430070, China. Tel: +86-27-8728 5016; Fax: +86-27-8728 2608;

E-mail: [zling604@yahoo.com](mailto:zling604@yahoo.com)

This file includes: Fig. S1-S2 and Supplemental Table1

Supplementary Figure Legends

**Figure S1.** **Meta-analysis of specific microbiome associated with antibiotic treatment, related to Figure 1.** Three-week-old female ICR mice (n=10) were orally provided with a cocktail of antibiotic (Abx) treatment, or administrated with sterile water as control. The feces were collected at 28 days post Abx treatment and 16S rRNA gene sequencing was performed to confirm whether gut microbiome was depleted by Abx-treatment. (A-C) Composition of microbiota in untreated or Abx-treated mice (Untreated mice, n=10, Abx-treated mice, n=10). (A) Relative abundance of specific bacterial phyla in untreated and Abx-treated mice. (B, C) Alpha diversity and beta diversity of the bacterial community. Sobs index based on Wilcoxon Rank-Sum Test at the OTU level and principal coordinate analyses (PCoA) based on Bray-Curtis dissimilarity illustrates the composition of stool microbiota in untreated and Abx-treated mice. (D) The Wilcoxon Rank-Sum Test demonstrates the relative abundance of bacterial taxa at the species level was significantly different between Abx-treated and untreated mice. (**P < 0.01; ***P < 0.001). (E) LEfSe analysis revealed that the relative abundance of 41 taxa of bacteria was significantly different between the Abx-treated and untreated mice at the different taxonomic levels (LDA>4, P < 0.05).

**Figure S2. Bacterial loads in stool samples post vaccination, related to Figure 1.** Abx-treated and untreated mice were intramuscularly inoculated with 10^7^ FFU rabies vaccine strain iLBNSE, and the stool samples were collected at the indicated times for 16S rRNA quantification by qPCR. Error bars in the graphs represent standard error (Untreated mice, n=10, Abx-treated mice, n=10). (***P < 0.001; ****P < 0.0001; Student’s *t* test).

**Figure S1.**


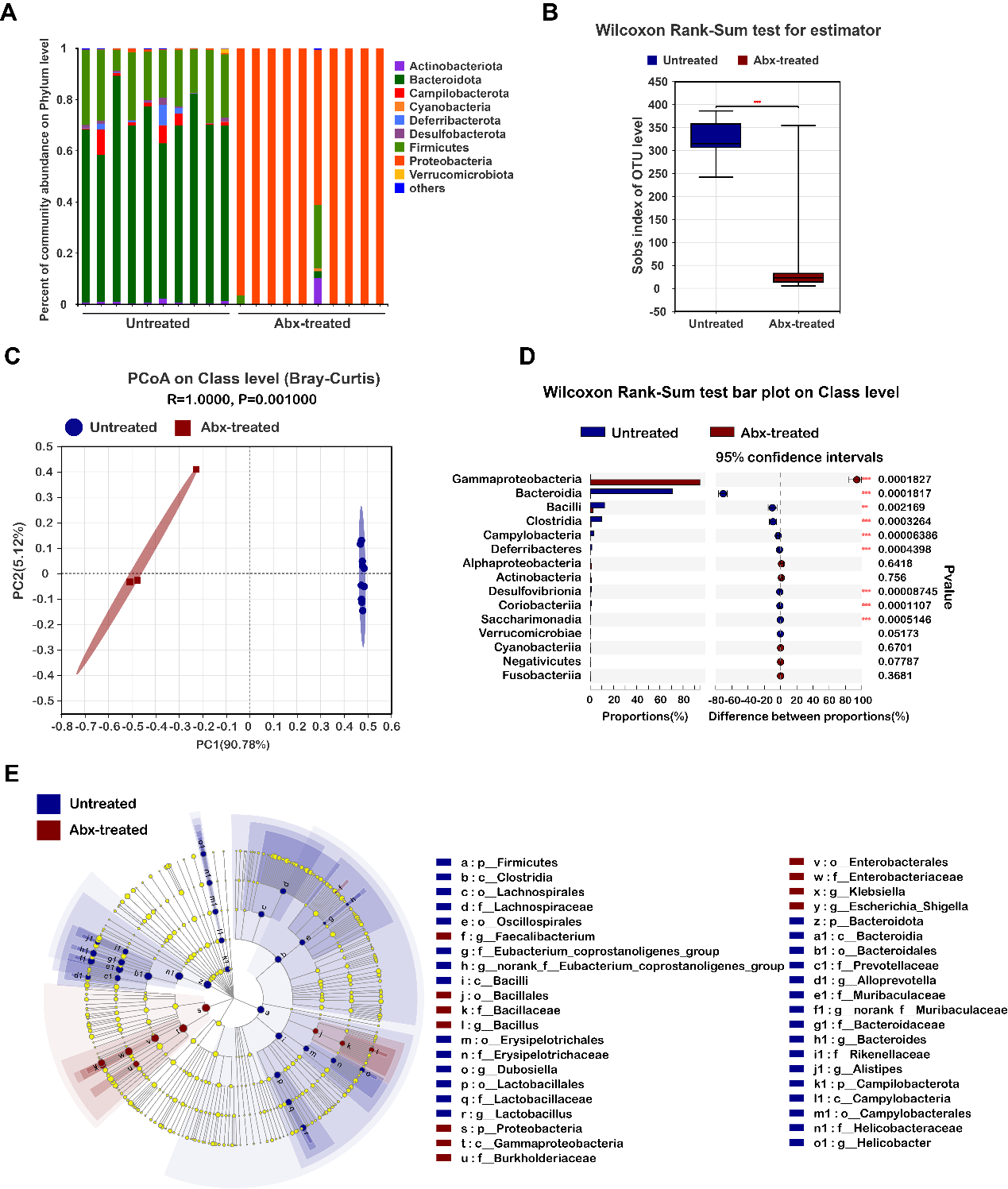


**Figure S2.**


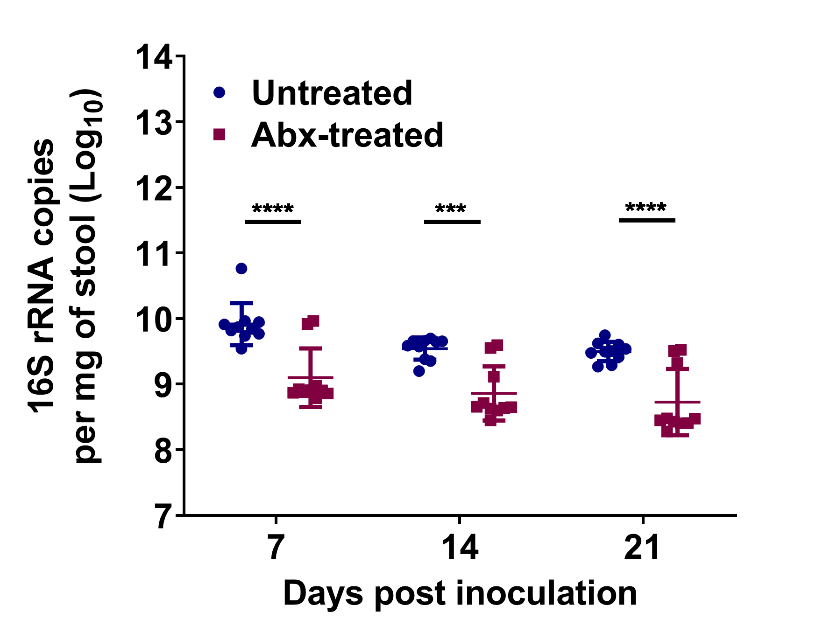


Supplementary Tables

**Table S1 Virus-neutralizing antibody (VNA) titers of mice immunized with rabies vaccines at indicated time points, related to Figure 6A.**

| No. | Mouse ID | VNA titer (IU/ml) | | |
| --- | --- | --- | --- | --- |
|  |  | **7 dpi** | **14 dpi** | **21 dpi** |
| 1 | C21 | 4.50 | 7.79 | 4.50 |
| 2 | C22 | 0.29 | 4.50 | 2.26 |
| 3 | C23 | 0.87 | 4.50 | 4.50 |
| 4 | C24 | 0.50 | 1.50 | 4.50 |
| 5 | C25 | 1.50 | 2.60 | 2.26 |
| 6 | C26 | 0.50 | 1.50 | 4.50 |
| 7 | C27 | 4.50 | 4.50 | 2.26 |
| 8 | C28 | 0.50 | 2.60 | 2.26 |
| 9 | C29 | 0.29 | 2.60 | 2.26 |
| 10 | C30 | 0.87 | 4.50 | 4.50 |
| 11 | C31 | 0.87 | 4.50 | 4.50 |
| 12 | C32 | 1.50 | 2.60 | 1.50 |
| 13 | C33 | 0.17 | 1.50 | 4.50 |
| 14 | C34 | 0.87 | 1.50 | 2.26 |
| 15 | C35 | 0.50 | 1.50 | 7.79 |
| 16 | C36 | 0.29 | 2.60 | 2.26 |
| 17 | C37 | 1.50 | 2.60 | 7.79 |
| 18 | C38 | 0.50 | 1.50 | 2.26 |
| 19 | C39 | 0.87 | 1.50 | 4.50 |
| 20 | C40 | 0.29 | 0.87 | 1.50 |
| 21 | SL26 | 0.66 | 1.50 | 1.97 |
| 22 | SL27 | 3.42 | 10.26 | 10.26 |
| 23 | SL28 | 1.50 | 5.92 | 5.92 |
| 24 | SL29 | 0.87 | 7.79 | 7.79 |
| 25 | SL30 | 1.97 | 5.92 | 5.92 |
| 26 | SL31 | 1.50 | 5.92 | 4.50 |
| 27 | SL32 | 1.50 | 13.50 | 7.79 |
| 28 | SL33 | 3.42 | 17.77 | 17.77 |
| 29 | SL34 | 1.50 | 5.92 | 4.50 |
| 30 | SL35 | 1.97 | 5.92 | 5.92 |
| 31 | SL36 | 3.42 | 17.77 | 10.26 |
| 32 | SL37 | 1.97 | 5.92 | 5.92 |
| 33 | SL38 | 1.14 | 5.92 | 4.50 |
| 34 | SL39 | 1.50 | 1.50 | 1.97 |
| 35 | SL40 | 0.22 | 0.87 | 0.66 |
| 36 | SL41 | 0.87 | 5.92 | 5.92 |
| 37 | SL42 | 0.22 | 1.97 | 1.50 |
| 38 | SL43 | 0.02 | 0.02 | 0.38 |
| 39 | SL44 | 0.29 | 0.87 | 1.50 |
| 40 | SL45 | 0.29 | 1.97 | 1.97 |
| 41 | SL46 | 0.17 | 2.60 | 1.97 |
| 42 | SL47 | 0.87 | 5.92 | 5.92 |
| 43 | SL48 | 1.14 | 7.79 | 4.50 |
| 44 | SL49 | 0.29 | 2.60 | 3.42 |
| 45 | SL50 | 0.22 | 0.87 | 1.14 |
| 46 | SL51 | 0.07 | 1.50 | 1.97 |
| 47 | SL52 | 3.42 | 13.50 | 17.77 |
| 48 | SL53 | 0.87 | 5.92 | 4.50 |
| 49 | SL54 | 1.14 | 4.50 | 5.92 |
| 50 | SL55 | 1.97 | 5.92 | 3.42 |
| 51 | SL56 | 1.14 | 5.92 | 3.42 |
| 52 | SL57 | 0.87 | 3.42 | 5.92 |
| 53 | SL58 | 0.07 | 0.50 | 1.50 |
| 54 | SL59 | 0.29 | 0.87 | 1.50 |
| 55 | SL60 | 1.97 | 7.79 | 4.50 |
| 56 | SL61 | 0.87 | 3.42 | 3.42 |
| 57 | SL62 | 0.50 | 1.14 | 1.97 |
| 58 | SL63 | 0.50 | 3.42 | 1.97 |
| 59 | SL64 | 0.29 | 1.97 | 1.50 |
| 60 | SL65 | 1.14 | 3.42 | 3.42 |
| 61 | SL66 | 1.50 | 5.92 | 5.92 |
| 62 | SL67 | 1.14 | 5.92 | 3.42 |
| 63 | SL68 | 4.50 | 17.77 | 13.50 |
| 64 | SL69 | 0.87 | 5.92 | 1.97 |
| 65 | SL70 | 1.50 | 4.50 | 2.60 |
| 66 | SL71 | 0.07 | 0.17 | 0.38 |
| 67 | SL72 | 0.38 | 0.87 | 0.87 |
| 68 | SL73 | 0.50 | 2.60 | 4.50 |
| 69 | SL74 | 1.14 | 3.42 | 3.42 |
| 70 | SL75 | 1.50 | 5.92 | 3.42 |
| 71 | SL76 | 0.87 | 3.42 | 3.42 |
| 72 | SL77 | 0.07 | 0.22 | 0.13 |
| 73 | SL78 | 3.42 | 17.77 | 10.26 |
| 74 | SL79 | 0.29 | 5.92 | 5.92 |
| 75 | SL80 | 4.50 | 17.77 | 5.92 |
| 76 | SL81 | 0.17 | 0.22 | 1.50 |
| 77 | SL82 | 0.50 | 1.97 | 1.97 |
| 78 | SL83 | 0.29 | 5.92 | 5.92 |
| 79 | SL84 | 0.38 | 3.42 | 3.42 |
| 80 | SL85 | 0.50 | 1.14 | 0.87 |
| 81 | SL86 | 0.38 | 1.14 | 1.97 |
| 82 | SL87 | 0.29 | 5.92 | 3.42 |
| 83 | J94 | 0.17 | 0.66 | 0.50 |
| 84 | D555 | 0.5 | 0.87 | 0.87 |
| 85 | D553 | 0.5 | 4.5 | 1.97 |
| 86 | D554 | 1.97 | 3.42 | 4.5 |
| 87 | D556 | 0.87 | 4.5 | 4.5 |
| 88 | D557 | 0.29 | 0.66 | 1.5 |
| 89 | D558 | 0.29 | 4.5 | 7.79 |
| 90 | D559 | 0.38 | 4.5 | 7.79 |
| 91 | D560 | 0.38 | 1.5 | 1.5 |
| 92 | D561 | 0.5 | 0.87 | 1.5 |
| 93 | D562 | 2.6 | 4.5 | 4.5 |
| 94 | D563 | 0.29 | 1.5 | 1.5 |
| 95 | D564 | 0.29 | 1.97 | 2.6 |
| 96 | D565 | 0.87 | 4.5 | 3.42 |
| 97 | D566 | 0.22 | 1.97 | 2.6 |
| 98 | D567 | 0.5 | 4.5 | 4.5 |
| 99 | D568 | 0.87 | 4.5 | 4.5 |
| 100 | D569 | 1.5 | 4.5 | 4.5 |
| 101 | D570 | 0.22 | 1.5 | 4.5 |
| 102 | D571 | 0.22 | 1.14 | 1.5 |
| 103 | D572 | 0.5 | 4.5 | 4.5 |
| 104 | D573 | 0.38 | 1.5 | 0.87 |
| 105 | J88 | 0.5 | 1.5 | 1.5 |
| 106 | J89 | 0.29 | 0.87 | 1.5 |
| 107 | J90 | 1.97 | 4.5 | 5.92 |
| 108 | J91 | 0.5 | 1.14 | 1.5 |
| 109 | J92 | 1.5 | 4.5 | 4.5 |
| 110 | J93 | 0.29 | 1.97 | 1.5 |
| 111 | J95 | 0.5 | 3.42 | 1.5 |
| 112 | J96 | 0.87 | 1.97 | 4.5 |
| 113 | H139 | 1.14 | 5.92 | 7.79 |
| 114 | H140 | 0.17 | 1.50 | 1.50 |
| 115 | H141 | 0.50 | 3.42 | 4.50 |
| 116 | H142 | 3.42 | 13.50 | 13.50 |
| 117 | H143 | 0.50 | 2.60 | 1.97 |
| 118 | H144 | 0.87 | 4.50 | 10.26 |
| 119 | H145 | 2.60 | 13.50 | 13.50 |
| 120 | H326 | 2.60 | 7.79 | 5.92 |
| 121 | H327 | 0.66 | 1.50 | 1.50 |
| 122 | H328 | 1.14 | 2.60 | 3.42 |
| 123 | H329 | 0.87 | 4.50 | 5.92 |
| 124 | H330 | 0.22 | 1.50 | 0.87 |
| 125 | H331 | 0.38 | 3.42 | 5.92 |
| 126 | H332 | 0.50 | 4.50 | 4.50 |
| 127 | H333 | 0.66 | 2.60 | 4.50 |
| 128 | H334 | 1.14 | 4.50 | 1.97 |
| 129 | H335 | 4.50 | 17.77 | 13.50 |
| 130 | H336 | 1.50 | 4.50 | 4.50 |
| 131 | H337 | 0.50 | 2.60 | 4.50 |
| 132 | H338 | 0.50 | 2.60 | 2.60 |
| 133 | H339 | 1.97 | 10.26 | 10.26 |
| 134 | H340 | 1.50 | 10.26 | 4.50 |
| 135 | H341 | 3.42 | 13.50 | 13.50 |
| 136 | H342 | 0.50 | 1.50 | 2.60 |
| 137 | H343 | 0.38 | 3.42 | 2.60 |
| 138 | H344 | 0.22 | 1.14 | 1.50 |
| 139 | H345 | 0.87 | 2.60 | 4.50 |
| 140 | H346 | 3.42 | 13.50 | 7.79 |
| 141 | H347 | 0.87 | 5.92 | 5.92 |
| 142 | H348 | 0.38 | 3.42 | 1.97 |
| 143 | H349 | 0.22 | 0.66 | 4.50 |
| 144 | H350 | 0.50 | 2.60 | 4.50 |
| 145 | H351 | 0.87 | 13.50 | 10.26 |
| 146 | H352 | 0.38 | 2.60 | 2.60 |
| 147 | H353 | 1.14 | 4.50 | 4.50 |
| 148 | H354 | 0.87 | 3.42 | 1.50 |
| 149 | H355 | 0.87 | 13.50 | 5.92 |
| 150 | H376 | 0.50 | 4.50 | 4.50 |
| 151 | H377 | 0.50 | 1.50 | 3.42 |
| 152 | H378 | 0.87 | 1.97 | 3.42 |
| 153 | H379 | 0.50 | 2.60 | 2.60 |
| 154 | H380 | 0.50 | 3.42 | 2.60 |
| 155 | H381 | 0.87 | 4.50 | 3.42 |
| 156 | H382 | 0.38 | 2.60 | 4.50 |
| 157 | H383 | 3.42 | 23.38 | 17.77 |
| 158 | H384 | 3.42 | 13.50 | 13.50 |
| 159 | H385 | 0.50 | 2.60 | 2.60 |
| 160 | H386 | 1.14 | 5.92 | 5.92 |
| 161 | H387 | 0.87 | 10.26 | 7.79 |
| 162 | H388 | 1.50 | 4.50 | 4.50 |
| 163 | H389 | 1.14 | 4.50 | 4.50 |
| 164 | H390 | 0.87 | 4.50 | 5.92 |
| 165 | H391 | 4.50 | 13.50 | 23.38 |
| 166 | H392 | 0.87 | 3.42 | 3.42 |
| 167 | H393 | 0.66 | 4.50 | 4.50 |
| 168 | H394 | 0.50 | 2.60 | 3.42 |
| 169 | H395 | 3.42 | 13.50 | 10.26 |
| 170 | H396 | 0.50 | 3.42 | 4.50 |
| 171 | H397 | 1.97 | 7.79 | 13.50 |
| 172 | H398 | 2.60 | 13.50 | 7.79 |
| 173 | H399 | 3.42 | 17.77 | 13.50 |
| 174 | H400 | 0.87 | 13.50 | 13.50 |
